# Comparative genomic analysis of inbred rat strains reveals the existence of ancestral polymorphisms

**DOI:** 10.1007/s00335-020-09831-7

**Published:** 2020-03-12

**Authors:** Hyeonjeong Kim, Minako Yoshihara, Mikita Suyama

**Affiliations:** Division of Bioinformatics, Medical Institute of Bioregulation, Kyushu University, Maidashi 3-1-1, Higashi-ku, Fukuoka, 812-8582 Japan

## Abstract

In an alignment of closely related genomic sequences, the existence of discordant mutation sites, which do not reflect the phylogenetic relationship of the genomes, is often observed. Although these discordant mutation sites are thought to have emerged by ancestral polymorphism or gene flow, their frequency and distribution in the genome have not yet been analyzed in detail. Using the genome sequences of all protein coding genes of 25 inbred rat strains, we analyzed the frequency and genome-wide distribution of the discordant mutation sites. From the comparison of different substrains, it was found that these loci are not substrain specific, but are common among different groups of substrains, suggesting that the discordant sites might have mainly emerged through ancestral polymorphism. It was also revealed that the discordant sites are not uniformly distributed along chromosomes, but are concentrated at certain genomic loci, such as *RT1*, major histocompatibility complex of rats, and olfactory receptors, indicating that genes known to be highly polymorphic tend to have more discordant sites. Our results also showed that loci with a high density of discordant sites are also rich in heterozygous variants, even though these are inbred strains.

**Electronic supplementary material:**

The online version of this article (10.1007/s00335-020-09831-7) contains supplementary material, which is available to authorized users.

## Introduction

After genome sequencing had been completed for representative model organisms, such as mice and rats (Waterston et al. 2002; Gibbs et al. 2004), genomes of other species have been sequenced and even more sequencing projects are in progress (Koepfli et al. 2015). At the beginning, genome comparison was conducted to elucidate the genes in a genome, because the basic gene set was thought to be conserved against the intergenic regions in a genome (Brenner et al. 1993). By comparing various genome sequences of different species in a clade, it is also possible to identify functionally important sites other than genes (i.e., conserved non-coding sequences). Not only genome sequence conservation among species but also species-specific presence or absence of certain genome sequences have provided insights into the characteristic traits of a species.

In the past decade, genomes of different strains or individuals within a species have been actively sequenced (Saar et al. 2008; Keane et al. 2011; Yalcin et al. 2012; Auton et al. 2015; Hermsen et al. 2015).Technically, this was facilitated for two main reasons. One reason is that, if the reference genome for a species has already been determined, genome sequencing for other strains for the species is comparatively easy because laborious assembling process is not required. The other reason is that next-generation sequencing technology, which is still drastically improving in terms of not only throughput but also time and cost, is greatly accelerating research in this direction. By comparing these closely related genome sequences, we can identify strain-specific traits such as disease susceptibility (e.g., Fairfield et al. 2011).

Rats are one of the species for which the genome sequence information of various strains is available (Saar et al. 2008; Hermsen et al. 2015). Compared to mice, in which genetic manipulation, such as gene knockout techniques, is well developed, rats have been, until recently, inferior with regard to genetic analyses, although rats are well suited, for example, for transplantation experiments and behavioral analyses because of their body size and obedient nature. The advent of genome editing technologies, however, has enabled the genetic manipulation of rats (Mashimo et al. 2013), and genetic analyses in rats will be greatly advanced in the future. Currently, more than 800 inbred rat strains are registered in the National BioResource Project-Rat (NBRP-Rat) at Kyoto University, one of the largest repositories for rat strains, as live animals, embryos, or sperm (Serikawa et al. 2009), and we have determined the protein coding genes and non-coding conserved sequences of some representative rat strains registered in NBRP-Rat (Yoshihara et al. 2016a, b; Kuramoto et al. 2017).

From the genome sequence comparison of such closely related strains, it is possible not only to identify strain-specific mutations to explain a certain trait for the strain, based on the differences in the genomic sequences, but also to conduct a large-scale analysis of mutation patterns among strains. A discordant mutation is one such mutation pattern, in which the pattern of base changes at a certain site in the genome is not consistent with the branching topology of the phylogenetic tree of strains based on the total number of variations among the strains (Fitch 1977). These discordant mutation patterns are thought to be observed if ancestral polymorphism or gene flow exists in a certain group of strains (Liu et al. 2008). Although it is possible to infer the reason why such mutation sites have emerged, their frequency and distribution in the genome are largely unknown.

In this study, using the genomic sequences of all protein coding genes of 25 inbred rat strains that we recently sequenced (Yoshihara et al. 2016a, b; Kuramoto et al. 2017), we first analyzed the phylogenetic relationship among them, and then comprehensively identified variant sites that showed discordant mutation patterns. We found that discordant sites are not uniformly distributed along chromosomes, but are concentrated at certain genomic loci. From detailed analyses, it is suggested that the discordant sites might have emerged mainly through ancestral polymorphism, and the loci are still rich in heterozygous variants, even though these are inbred strains.

## Materials and methods

### Data set

We used the genomic sequences corresponding to the coding regions of 25 inbred rat strains (DDBJ Sequence Read Archive accession number: DRA004543; DDBJ/EMBL/GenBank accession number: PRJDB4648) obtained from our previous studies (Yoshihara et al. 2016a, b; Kuramoto et al. 2017) (Table 1). These 25 strains were selected according to the following three categories: representative inbred strains (F344/DuCrlCrlj, F344/Jcl, F344/NSlc, and F344/Stm), those originating from wild populations (BN/SsNSlc, DOB/Oda, IS/Kyo, IS-*Tlk*/Kyo, LE/Stm, LEC/Tj, and NIG-III/Hok), and disease models derived from selective breeding (BDIX/NemOda, BDIX.Cg-*Tal*/NemOda, BUF/MNa, HTX/Kyo, HWY/Slc, KFRS3B/Kyo, KFRS4/Kyo, NER/Kyo, PVG/Seac, RCS/Kyo, WTC/Kyo, WTC-*swh*/Kyo, ZF, and ZFDM). All these strains are kept in NBRP-Rat, Kyoto University (Kyoto, Japan) (Serikawa et al. 2009). We downloaded rat genome rn5 (RGSC 5.0, March 2012) from Ensembl (ftp://ftp.ensembl.org/pub/release-79/fasta/rattus_norvegicus/dna/) (Zerbino et al. 2018) and used this as a reference genome.

**Table 1:** List of 25 rat strains analyzed in this study

Rat no	Strain name	Inbred generations^a^	NBRP no
1	BDIX.Cg-*Tal*/NemOda	-	0305
2	BDIX/NemOda	F11 (March 2012)	0304
3	BN/SsNSlc	-	0149
4	BUF/Mna	F122 (April 2012)	0200
5	DOB/Oda	F29 (April 2012)	0307
6	F344/DuCrlCrlj	-	0506
7	F344/Jcl	-	None
8	F344/NSlc	F188	0156
9	F344/Stm	F91 (April 2012)	0140
10	HTX/Kyo	F? + 54 (March 2012)	0006
11	HWY/Slc	F? + 11	0152
12	IS/Kyo	F88 (March 2012)	0008
13	IS-*Tlk*/Kyo	F65 (May 2009)	0009
14	KFRS3B/Kyo	-	0571
15	KFRS4/Kyo	F24 (April 2012)	0572
16	LE/Stm	F111 (April 2012)	0139
17	LEC/Tj	F100	0051
18	NER/Kyo	F70 (March 2012)	0010
19	NIG-III/Hok	F140 (April 2012)	0044
20	PVG/Seac	F59 (April 2012)	0080
21	RCS/Kyo	F47 (March 2012)	0011
22	WTC/Kyo	F86 (March 2012)	0020
23	WTC-*Swh*/Kyo	F37 (March 2012)	0287
24	ZF	-	None
25	ZFDM	-	None

Information obtained from the NBPR-Rat web site (https://www.anim.med.kyoto-u.ac.jp/NBR/). The inbred generation represents the generation at the time point shown in the parentheses. For strains whose time point is not provided, it is not written. Question mark (“?”) indicates that the number of inbred generations before transfer to NBRP-Rat is unknown. Hyphen (“-”) indicates that the information about inbred generations was not available

### Identification and annotation of variants

The genomic sequence data corresponding to the coding regions were processed as reported in our previous study (Yoshihara et al. 2016a, b). In brief, the sequencing reads were mapped to the rat reference genome (rn5) using BWA (v.0.7.4) (Li and Durbin 2009) with the default parameters. SAMtools (v.0.1.12a) (Li et al. 2009; Li 2011), Picard Tools (v.1.87) (https://broadinstitute.github.io/picard/), and the Genome Analysis Toolkit (GATK, v.2.5.2) (McKenna et al. 2010) were used for post-processing of mapped reads. The UnifiedGenotyper utility in GATK was used for variant calling. Since there is a risk of misalignment of sequencing reads and collapsed mapping of sequencing reads, we used only homozygous variants, but no heterozygous variants in this study unless otherwise noted. The details of these conditions are illustrated in “Discussion”. ANNOVAR (version 2015-03-22) (Wang et al. 2010) was used to annotate these variants.

### Construction of a phylogenetic tree

Phylogenetic trees were constructed in the following three steps. (1) The distance matrix was cleated based on SNV data. (2) The distance matrix was supplied to the “ape” package (v.5.0) (Paradis and Schliep 2019) in R (v.3.5.1) (The R Project for Statistical Computing, Vienna, Austria) to create a phylogenetic tree based on the neighbor-joining method (Saitou and Nei 1987). The data of the phylogenetic tree was obtained in Newick format. (3) To visualize the Newick-formatted file obtained above, a phylogenetic tree was depicted by the Dendroscope 3 program (v.3.5.10) (Huson and Scornavacca 2012).

### Identification of discordant sites

To automatically identify discordant sites, we first generated multiple genome sequence alignment of the coding DNA sequences (CDSs) by reflecting the SNV data into the rat reference sequence. Information about CDSs was obtained from the Ensembl (Zerbino et al. 2018) and RefSeq (O’Leary et al. 2016) annotations in the UCSC Genome Browser (Casper et al. 2018). Then, discordant sites were identified as homozygous variant sites in the genome sequence alignment that showed patterns of mutation inconsistent with the branching order in the phylogenetic tree. More precisely, at a variant site in a closely related substrain cluster, if the same variation is also observed in other strains, which are thought to be more distantly related to those in the substrain cluster, we defined such a site as a discordant site. The examples of the discordant sites are illustrated in “Results”. At this step, we used the “tree” module of the ETE Toolkit 3 (v.3.1.1) (Huerta-Cepas et al. 2016) to analyze the branching pattern of the phylogenetic tree in Newick format. The alignments were visualized using Jalview (v.2) (Waterhouse et al. 2009).

To analyze the distribution of discordant sites along chromosomes, information on the chromosome lengths and cytogenetic bands of rats (rn5) was downloaded from the UCSC Genome Browser (https://hgdownload.cse.ucsc.edu/goldenPath/rn5/bigZips/rn5.chrom.size and https://hgdownload.cse.ucsc.edu/goldenPath/rn5/database/cytoBand.txt.gz, respectively). The graph of the chromosomal distribution of the discordant sites was created using the “ggplot2” package (Wickham 2016) of the R software.

### Enrichment analysis of genes

For enrichment analysis of genes, we used Metascape (Zhou et al. 2019). We set both “Input as species” and “Analysis as species” options to “*R. norvegicus*.”

## Results

### Identification of coding variants in each strain

First, we identified homozygous coding variants in the 25 inbred rat strains. This analysis was performed in comparison with the reference genome, which is determined for Brown Norway rats (Gibbs et al. 2004) (Table 1). The variants were then classified by applying ANNOVAR (Wang et al. 2010). In each strain, the number of synonymous variants was approximately twice that of non-synonymous variants. The number of stop-gain mutations was approximately 10 times as much as the number of stop-loss mutations (Tables 2, S1), showing a similar ratio to that reported in a large-scale mutational analysis in cattle (Charlier et al. 2016). The highest number of mutations was observed in the DOB/Oda strain, which is a Japanese wild-derived rat strain (Kuramoto et al. 2013).

**Table 2:** The numbers of synonymous, non-synonymous, stop-gain, and stop-loss SNVs in the 25 rat strains

Strain name	Synonymous SNV	Non-synonymous SNV	Stop-gain/loss
BDIX.Cg-*Tal*/NemOda	9006	4777	25/1
BDIX/NemOda	8659	4565	19/2
BN/SsNSlc	113	193	3/0
BUF/Mna	8518	4777	22/3
DOB/Oda	11,042	5810	28/2
F344/DuCrlCrlj	8396	4601	20/1
F344/Jcl	8283	4538	22/1
F344/NSlc	8294	4548	19/1
F344/Stm	8379	4547	20/1
HTX/Kyo	8496	4741	17/4
HWY/Slc	8680	4609	18/2
IS/Kyo	10,585	5820	31/2
IS-*Tlk*/Kyo	10,596	5819	30/2
KFRS3B/Kyo	8440	4480	21/2
KFRS4/Kyo	9019	5049	18/5
LE /Stm	8545	4558	18/4
LEC/Tj	9389	5103	26/3
NER/Kyo	7140	3829	11/3
NIG-III/Hok	9150	4960	23/3
PVG/Seac	9113	5013	21/4
RCS/Kyo	8421	4594	24/4
WTC/Kyo	10,017	5713	28/3
WTC-*Swh*/Kyo	10,018	5724	29/3
ZF	8395	4517	18/2
ZFDM	8486	4623	18/2

### Phylogenetic trees based on exome target capture SNV data

**Fig. 1:**
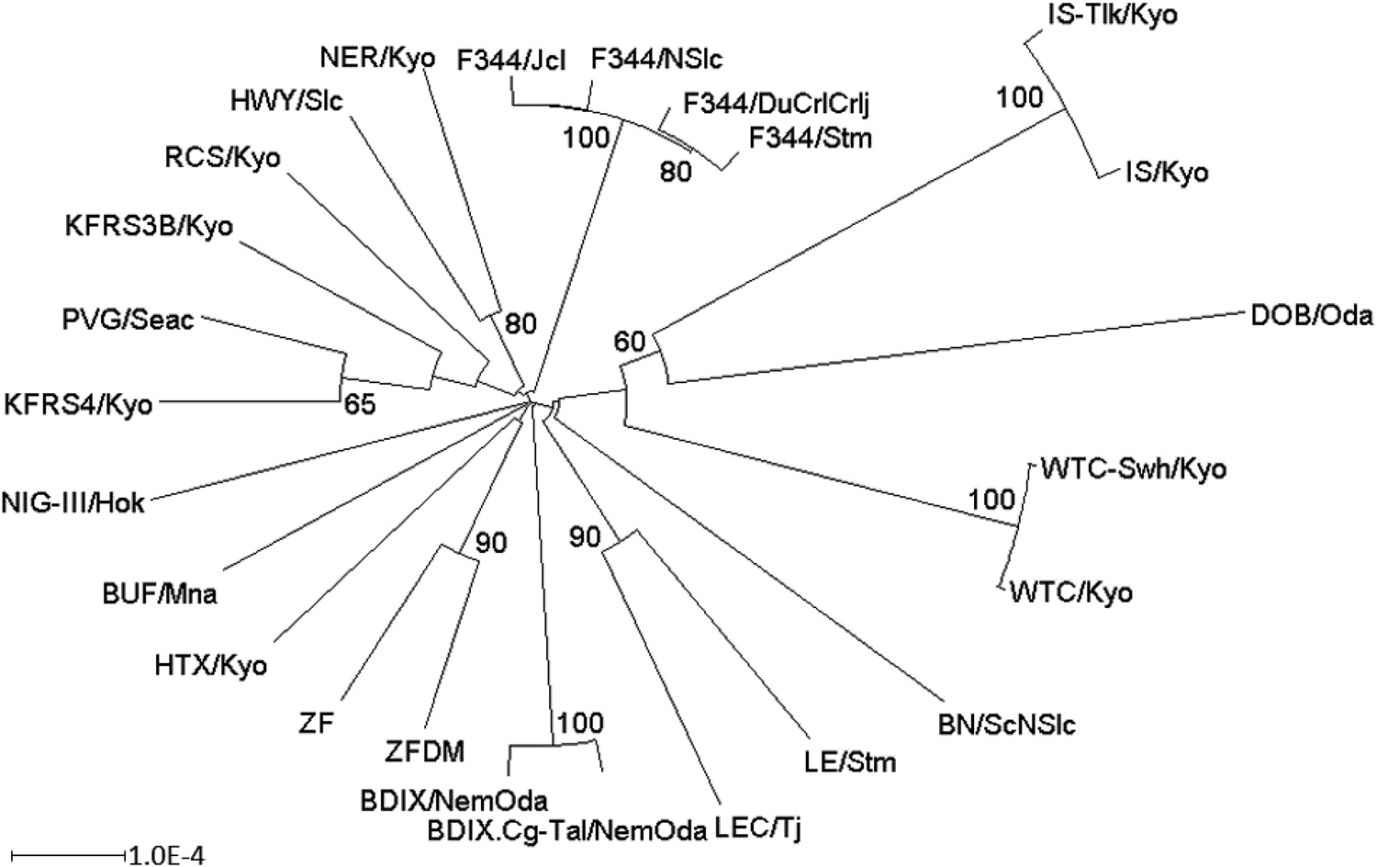
Phylogenetic tree of 25 inbred rat strains. This tree was obtained using the neighbor-joining algorithm based on SNV data of all the coding regions. The value at each internal branch indicates the branch support value as a percentage for chromosome level. Only values greater than 50% are shown. The scale bar at the bottom left indicates one nucleotide substitution per 10,000 bases

To reveal the genetic relationship of the 25 inbred rat strains (Table 1), we attempted to construct a phylogenetic tree. For this, we prepared a distance matrix that summarized the genetic distances of all possible pairs of strains. Here, the genetic distances are calculated on the basis of the information about the mutations compared to the reference genome. For example, in calculating the number of mutations between strains A and B from the information about the mutations of strain A versus the reference genome and strain B versus the reference genome, we enumerated only those mutations that can be observed either in strain A or in strain B but not in both strains. Then, the obtained distance matrix was subjected to the neighbor-joining algorithm to construct an unrooted phylogenetic tree (Fig. 1). As DOB/Oda is a wild-derived rat, it is clearly separated from the other laboratory rat strains (Kuramoto et al. 2013). A moderate similarity between PVG/Seac and KFRS4/Kyo well reflects the origin of the KFRS4/Kyo strain; that is, the strain is an inbred one derived from the crossing of a fancy rat with the PVG/Seac strain (Kuramoto et al. 2010). Using the same procedure, the phylogenetic tree was also made for each of the 20 rat autosomes (Supplementary Fig. S1). The percentage values on branches represent the branch support value at the chromosome level (Fig. 1). For example, 80% means that the phylogeny of 16 out of 20 autosomes has the same internal branch as the corresponding one in the phylogeny of all the chromosomes. There are clusters with a high branch support value, each of which is comprised of substrains: BDIX (BDIX/NemOda and BDIX.Cg-*Tal*/NemOda), F344 (F344/DuCrlCrlj, F344/Jcl, F344/NSl, and F344/Stm), IS (IS-*Tlk*/Kyo and IS/Kyo), WTC (WTC/Kyo and WTC-*swh*/Kyo), and ZF (ZF and ZFDM). In the phylogenetic tree for chromosome 20 (Supplementary Fig. S1), the LE/Stm strain is not directly clustered with the LEC/Tj strain, but is clustered with BN/ScNSlc and the BDIX cluster. The substrains in the ZF cluster, ZF and ZFDM, are also separated in the phylogenetic tree for chromosome 20. Such instability in branching might be due to the relatively small number of mutations on the relatively short chromosome 20. Separation of ZF substrains was also observed in the phylogenetic tree for chromosome 16 (Supplementary Fig. S1).

### Discordant sites among 25 rat strains

**Fig. 2:**
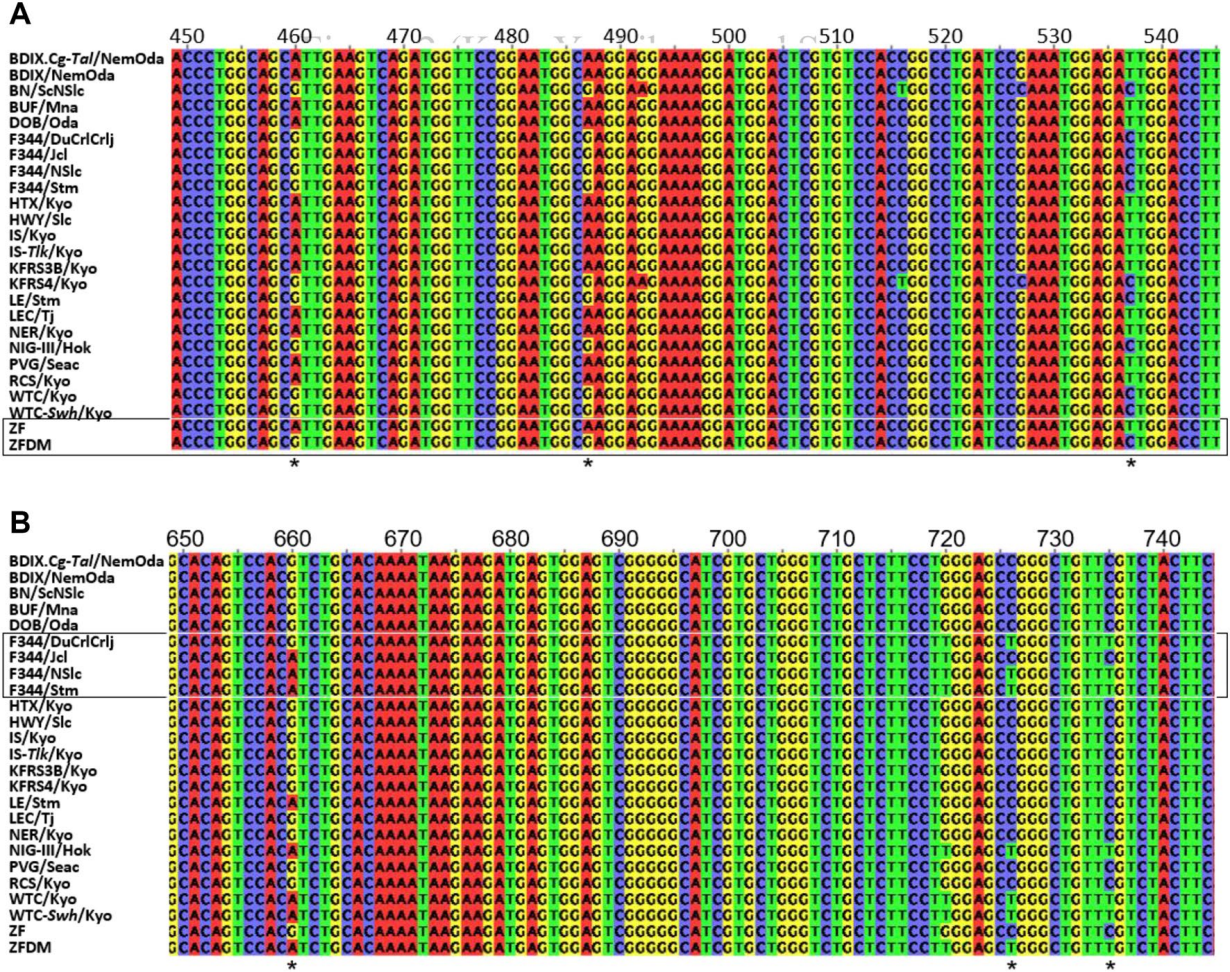
Examples of discordant sites in multiple sequence alignments of *RT1-Db1* gene on 20p12. The box indicates the cluster of substrains. The numbers above the alignment indicate the positions in mRNA. Discordant sites are indicated by asterisks under the alignments. **a** Discordant sites observed in the cluster of ZF substrains. **b** Discordant sites observed in the cluster of F344 substrains

By looking at multiple genome sequence alignments of these strains in detail, we were able to easily find variant sites that showed patterns of mutation inconsistent with the branching order in the phylogenetic tree constructed from the number of mutations in the coding regions. For example, variants observed in the cluster of ZF, which consists of the ZF and ZFDM strains, also existed in the other strains, despite their distance measured by the number of mutations in coding regions (Fig. 2a). Hereafter, we define such a variant site, i.e., the site with variants in a cluster of closely related substrains also existing in the other strains, as a “discordant site.” Discordant sites are also observed in another cluster of closely related substrains, such as the cluster of F344 (Fig. 2b), and in other genes (Supplementary Fig. S2).

To quantitatively assess the occurrence of discordant sites, we counted the number of discordant sites for each cluster of substrains (Tables 3, S2). The number of discordant sites is not uniform among the five clusters of substrains, but varies from 234 (for WTC) to 6146 (for ZF). This difference may be attributed to the divergence of the substrains in each cluster; that is, a more diverged cluster, such as ZF, tends to have a higher content of discordant sites, whereas a less diverged cluster, such as WTC, tends to have a lower content of discordant sites.

**Table 3:** The number of discordant sites in the CDS of five clusters of substrains (BDIX, F344, IS, WTC, and ZF)

Strain	The number of discordant sites
BDIX	1410
F344	2103
IS	824
WTC	234
ZF	6146

**Fig. 3:**
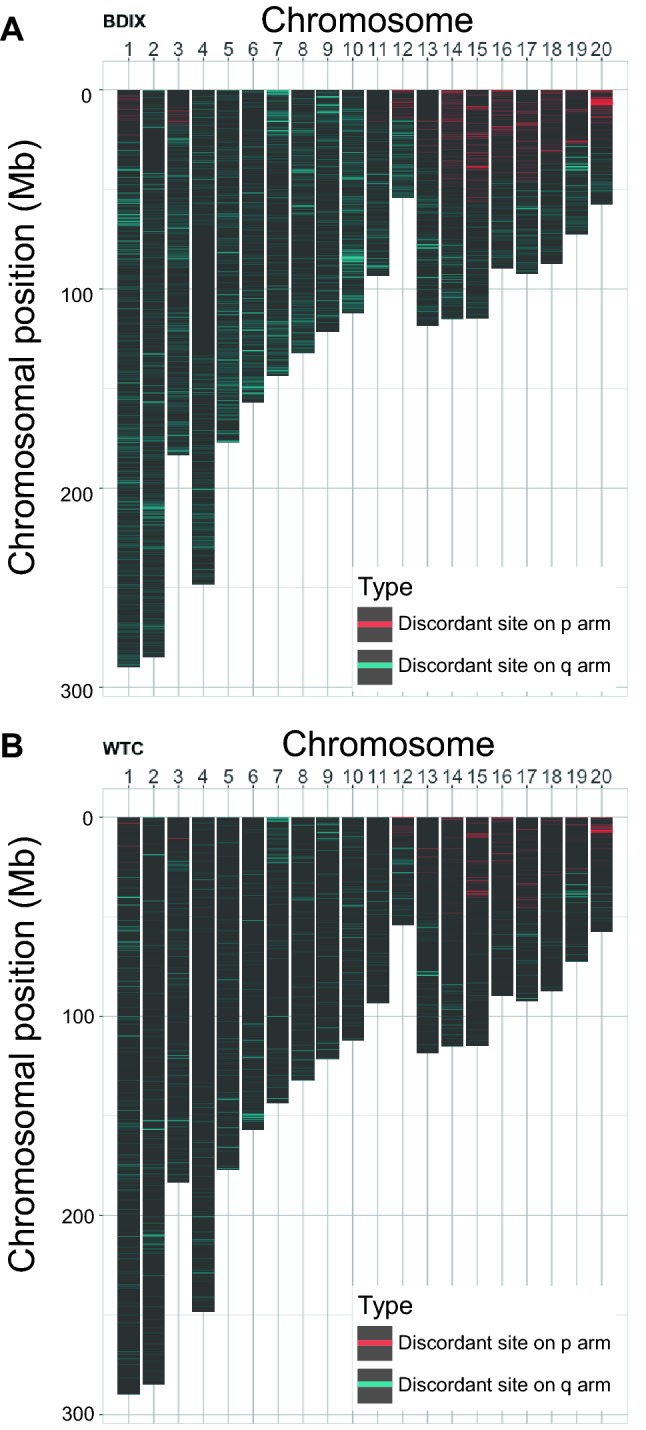
Chromosomal distribution of discordant sites in the autosomes of the clusters of **a** BDIX and **b** WTC substrains. The red and blue horizontal lines on the chromosomes represent discordant sites in the short (p) arm and long (q) arm, respectively

To analyze the distribution of the discordant sites along chromosomes, those sites were visualized by displaying them on bar graphs that represented ideograms of rat chromosomes (Fig. 3). Although discordant sites are observed all over the chromosomal positions, they are not uniformly distributed, but have a clear trend of aggregating at certain genomic locations. Moreover, the positions of these genomic regions, which have a relatively high number of discordant sites, are shared among different clusters of substrains (Fig. 3). For example, highly dense regions of discordant sites in the p-arm of chromosome 20 found in the BDIX substrains (Fig. 3a) are also found in the WTC substrains (Fig. 3b). The overall pattern of the distribution of discordant sites is also similar in other clusters of substrains (Supplementary Fig. S3), indicating the existence of certain characteristics for such regions.

### Enrichment analysis of discordant site abundant genes

To comprehensively analyze the characteristics of regions with a relatively high number of discordant sites, we first extracted genes with discordant sites and sorted them according to the density of the discordant sites. There were 6,216 genes with at least one common discordant site in the five clusters of substrains. The gene with the highest density of discordant sites was *RT1*, which is a major histocompatibility complex (MHC) of rats, located at 20p12 (Aptekman 1960; Günther and Walter 2001).

**Fig. 4:**
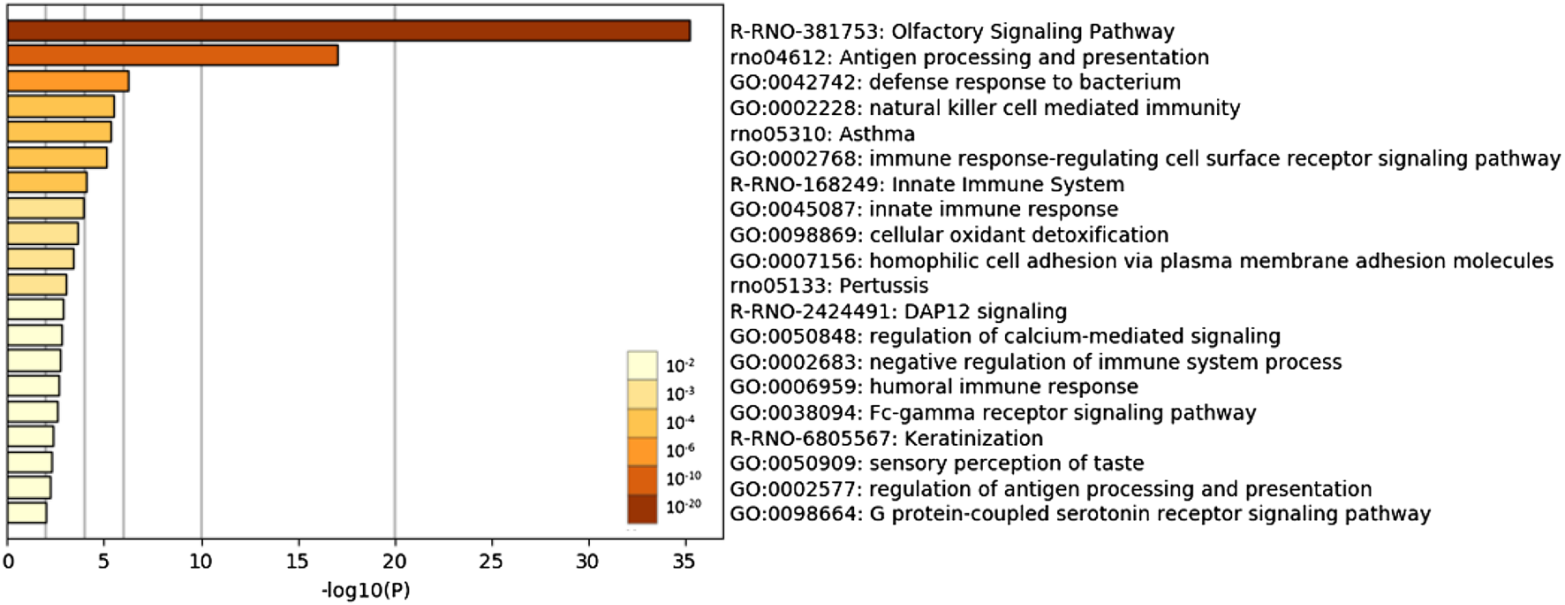
Bar graph of the functional enrichment analysis by Metascape. The enrichment analysis was performed using the top 500 genes from the list of genes with discordant sites. The bars are colored by *P*-values. GO, gene ontology; rno, KEGG pathway for rats; R-RNO, Reactome gene sets for rats

The top 500 genes with a high density of discordant sites were subjected to a functional enrichment analysis by Metascape (Zhou et al. 2019) (Supplementary Table S3). The result showed that discordant site-rich genes tend to be immune-related genes and olfactory receptor genes (Fig. 4). As the cutoff, the top 500, was arbitrarily selected, we tried some other cutoff values, that is, a standard deviation value of the discordant site content, which yielded 1,176 genes, and the mean value of the discordant site content, which yielded 2,035 genes (Supplementary Fig. S4). The functional enrichment analysis of these lists of genes also yielded similar results to that of the top 500 genes (Supplementary Fig. S5).

### Correspondence between discordant site-rich regions and heterozygous variant-rich regions

**Fig. 5:**
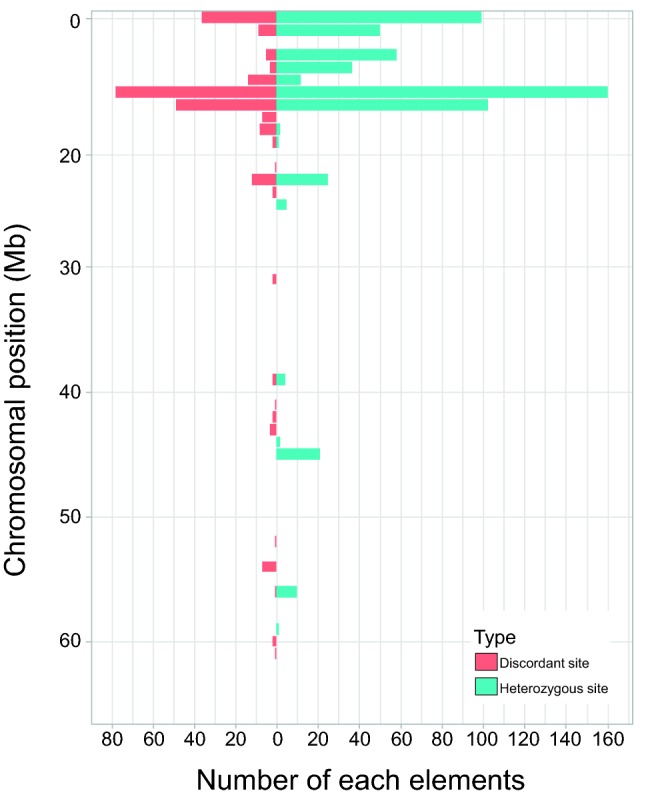
Distributions of discordant sites and heterozygous SNVs. The number of discordant sites and heterozygous SNVs in the F344 are shown in orange and blue, respectively, on chromosome 20

Discordant sites are concentrated on the genes that tend to be polymorphic, such as immune-related genes and genes involved in sensory signaling pathways. Although this might be reflected by a high level of polymorphism in their ancestors, the strains in the present state still might have a certain degree of polymorphism in the same regions, even though they are inbred strains. To confirm this, we enumerated the number of heterozygous sites. They have heterozygous sites up to 0.02% (in WTC-*Swh*/Kyo), this is comparable to what is expected for the inbred generations (F37) (Table 1). We did not observe any correlation between inbred generations and proportion of heterozygous variants. We plotted these heterozygous variations along the chromosomes and compared the distribution with that of the discordant sites (Fig. 5). Indeed, both distributions correspond well with each other, indicating that the regions where discordant sites are concentrated still have heterozygous sites.

## Discussion

Inbred strains are a valuable resource for analyzing the genetic bases of strain-specific traits. Comparative genomic sequence analysis among strains is a fundamental step for identifying genomic sequence segments responsible for certain traits and also for understanding the mode of genomic sequence divergence. For this, we conducted a comparative genomic sequence analysis of coding regions of 25 inbred rat strains, focusing on discordant sites, which show an inconsistency between genetic relatedness of strains and patterns of sequence variations. We found that such sites exist all over the chromosomes. They are not uniformly distributed along chromosomes, but are concentrated on the loci comprising immunity and sensory genes. In addition, we found that regions rich in discordant sites are also rich in heterozygous variants, although these strains are thought to be genetically homozygous for most of the loci because of inbreeding.

There are at least two possible explanations for the emergence of discordant sites. One possibility is the existence of ancestral polymorphisms (Slatkin and Pollack 2008). This can be explained as follows: during the establishment process for each inbred strain, polymorphic loci that existed in their ancestral strain have randomly fixed to a single allele. The other possibility is the result of gene flow (Slatkin 1987). Genomic segments that migrated from other strains could have brought variants that are inconsistent with the strains’ phylogenetic relationships. However, because gene flow is a stochastic process, it is not likely to happen in certain loci in multiple independent clusters of substrains. Accordingly, we concluded that discordant sites would have originated from polymorphic loci that existed in their nearest common ancestor. This idea can be supported by the fact that such genomic regions contain immunity and sensory genes, such as *RT1* and olfactory receptors, which are known to be highly polymorphic (Ehlers et al. 2000; Takagi et al. 2009). In addition, heterozygous variants are also enriched in those loci, indicating that such loci still have polymorphisms even though these are inbred strains. This observation further supports the idea that the discordant sites have emerged by ancestral polymorphisms.

A possible pitfall in the present study is that there could be a misalignment of sequence reads in genomic regions with very high similarities. If this happens, we may have false variants (Supplementary Fig. S6a). To prevent such instances, we adopted stringent criteria for read alignment, i.e., we used only those reads that were uniquely mapped to the reference genome with two or fewer mismatches. However, even using only uniquely mapped reads, we could still have false variants by copy number changes between the reference genome and the genome of a strain under consideration (Keane et al. 2011; Doran et al. 2016; Ramdas et al. 2019). For example, if there is a duplicated region in the genome of a strain under analysis, while the reference genome has only a single copy, then the sequencing reads that come from the duplicated region are forced to map to a single locus of the reference genome (Supplementary Fig. S6b). In this case, any differences between the duplicated regions should be detected as variants. To prevent such false variants, we only used homozygous variants for discordant site detection because such collapsed read mapping should result in heterozygous variants (Ramdas et al. 2019). With these conditions, we reliably obtained more high-quality alignments and variants.

In summary, we identified discordant sites by comparing the phylogenetic trees of inbred strains and each position in the genome alignment. Their emergence can be attributed to ancestral polymorphisms because of their enrichment in highly polymorphic loci, such as *RT1* (Takagi et al. 2009). These regions seem to be still heterozygous to some extent because heterozygous sites are also enriched in discordant site-rich regions. These findings are concordant with some previous reports for other inbred species (Lilue et al. 2018; Wang et al. 2019), providing valuable insights for understanding the genetic characteristics and diversity in highly polymorphic loci in the process of inbreeding. Indeed, in the process of naturally occurring inbreeding, such loci are thought to be under balancing selection and are shown to be heterozygous (Sato et al. 2002; Aguilar et al. 2004; Lins et al. 2018). Our results, together with the findings in other previously reported species (Lilue et al. 2018; Wang et al. 2019), suggest that balancing selection may also act on such loci in the process of artificial inbreeding.

## Electronic supplementary material

Below is the link to the electronic supplementary material.

Electronic supplementary material 1 (PDF 1932 kb)

Electronic supplementary material 2 (XLSX 12836 kb)

Electronic supplementary material 3 (XLSX 280 kb)

Electronic supplementary material 4 (XLSX 50 kb)
