# Supplementary material for: Comparative genomic analysis of inbred rat strains reveals the existence of ancestral polymorphisms
Source: Mamm Genome. 2020 Mar 12;31(3):86–94. doi: 10.1007/s00335-020-09831-7 (PMC7200647; doi:10.1007/s00335-020-09831-7)
Supplement: Supplementary file 1 — Electronic supplementary material 1 (PDF 1932 kb) [file 335_2020_9831_MOESM1_ESM.pdf]

Supplementary Figures

Title:

Comparative genomic analysis of inbred rat strains reveals the existence of ancestral polymorphisms

Journal name:

Mammalian genome

Authors:

Hyeonjeong Kim, Minako Yoshihara, Mikita Suyama*

*Corresponding author

Affiliation and Address:

Division of Bioinformatics, Medical Institute of Bioregulation, Kyushu University

Maidashi 3-1-1, Higashi-ku, Fukuoka 812-8582, Japan

Contact:

Mikita Suyama

E-mail: mikita@bioreg.kyushu-u.ac.jp

Phone: +81-92-642-6384

ORCID: 0000-0001-9526-3193

Chr1

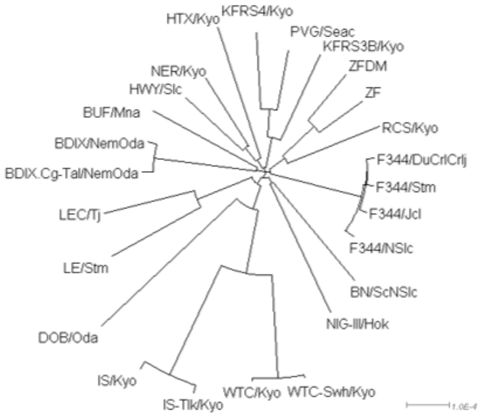

Chr2

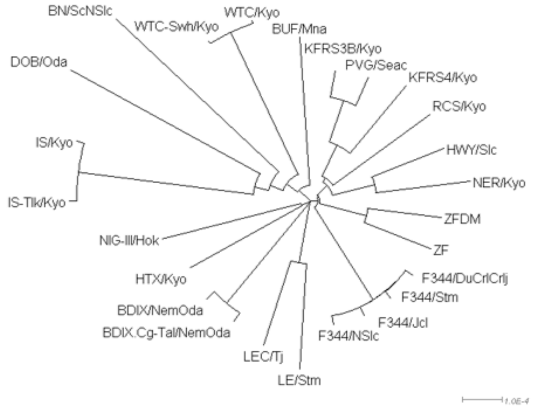

Chr3

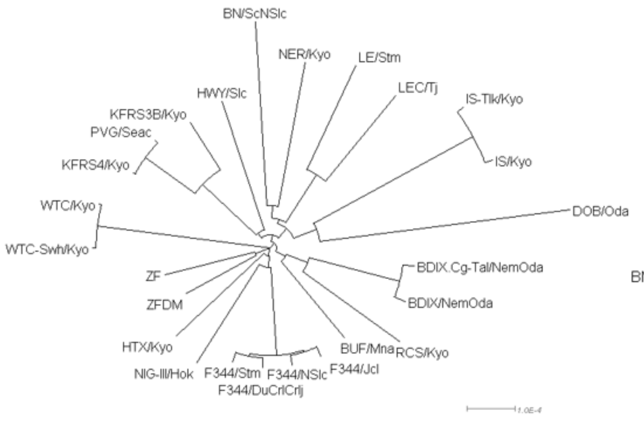

Chr4

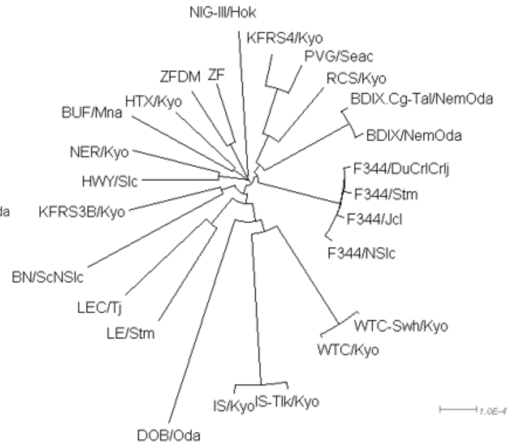

Chr5

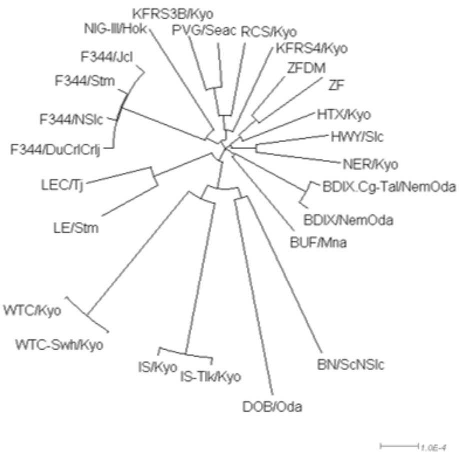

Chr6

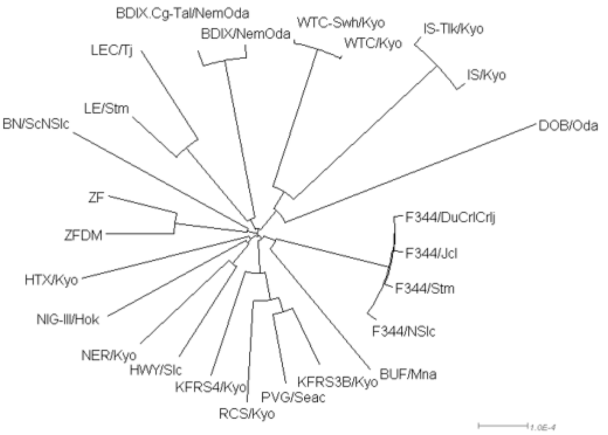

Chr7

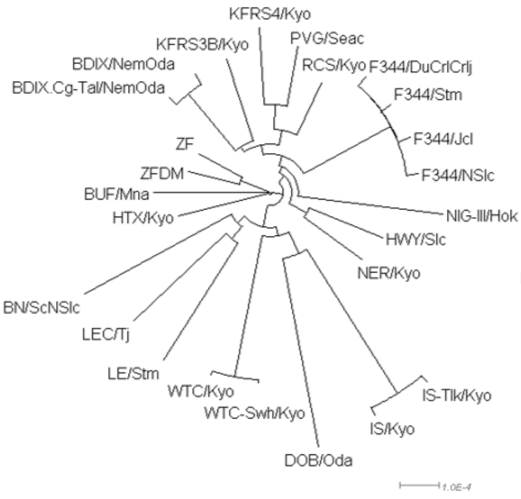

Chr8

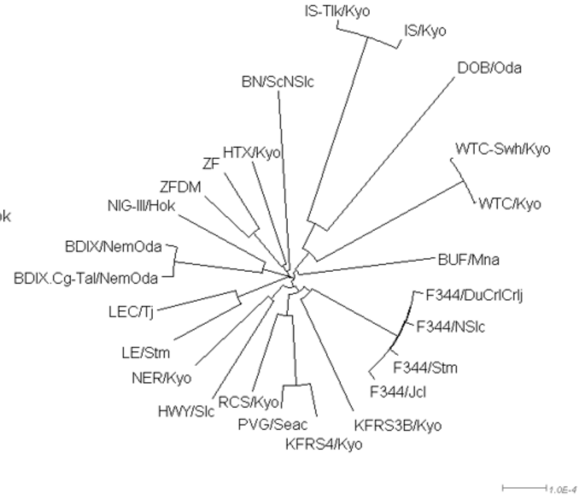

Chr9

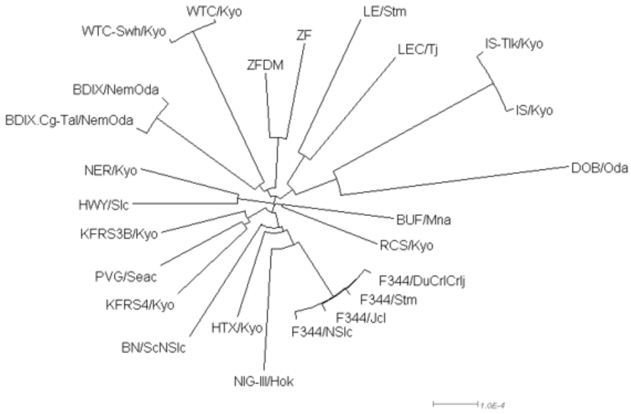

Chr10

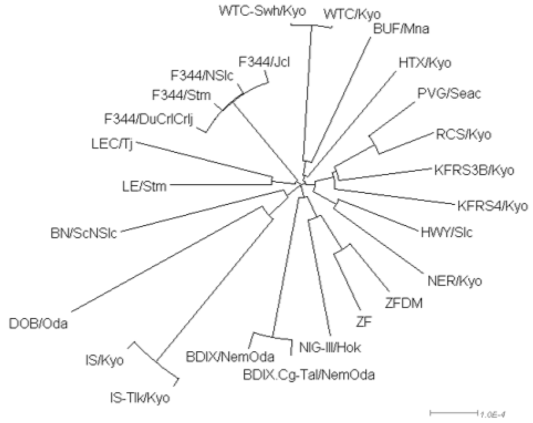

Chr11

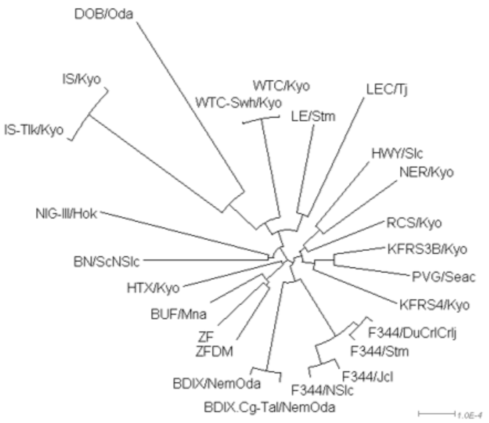

Chr12

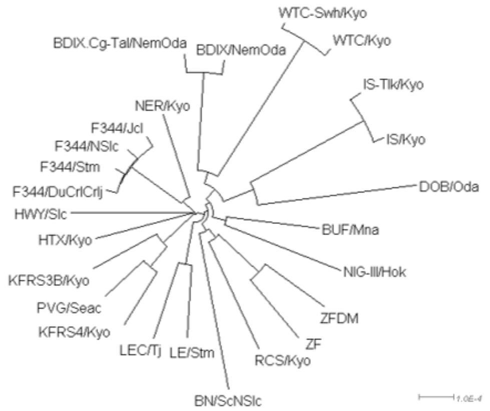

Chr13

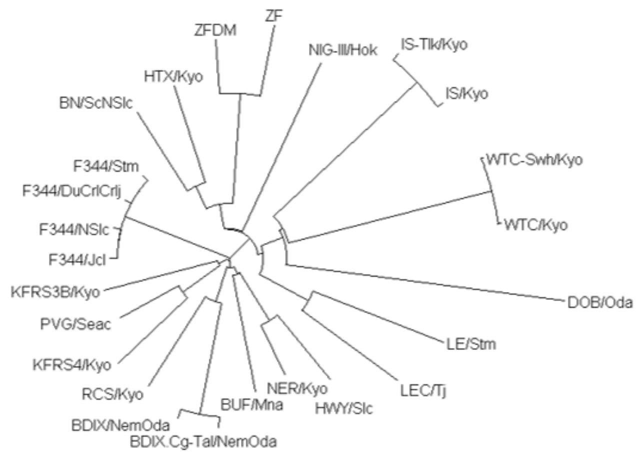

Chr14

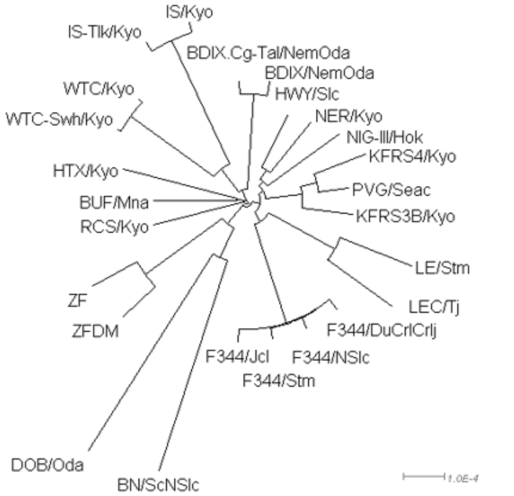

Chr15

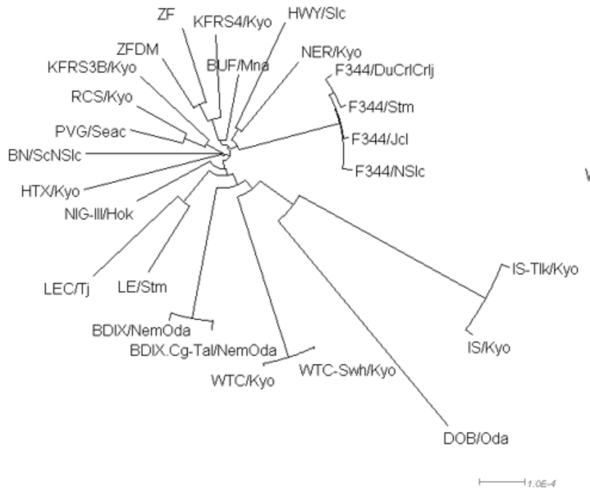

Chr16

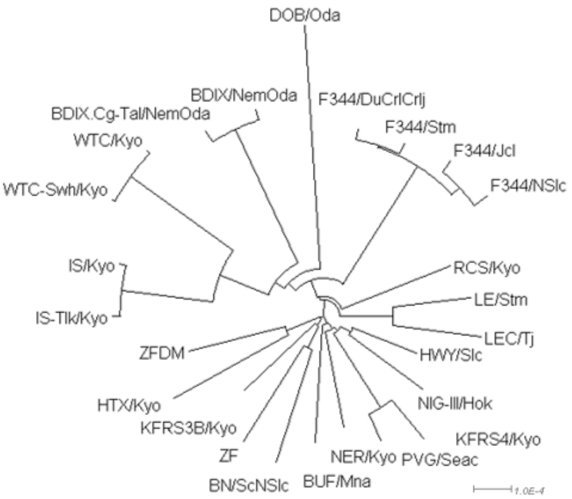

Chr17

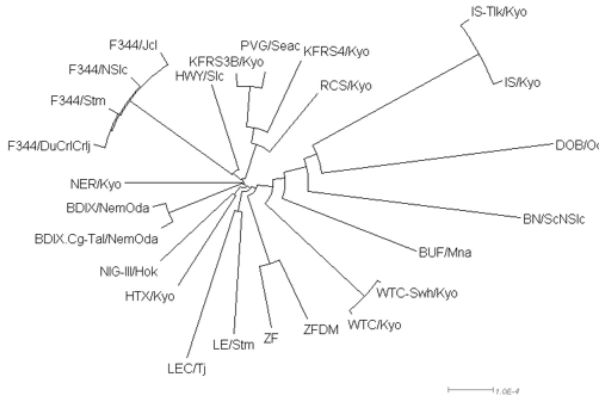

Chr18

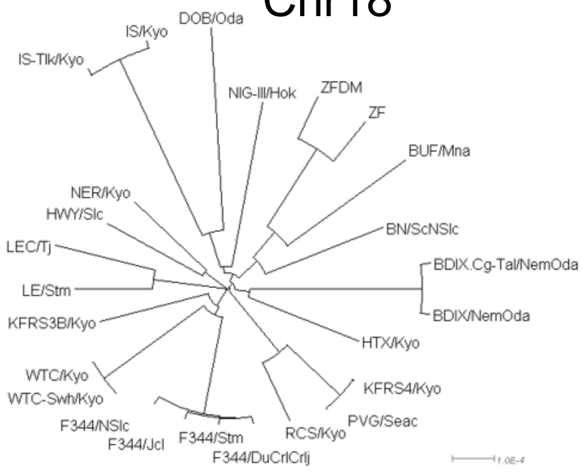

Chr19 Chr20

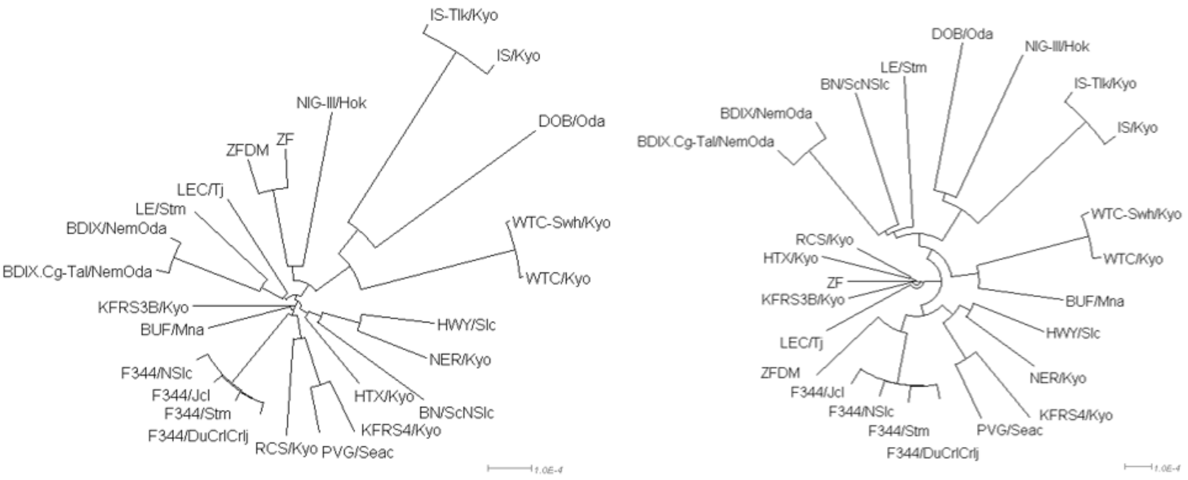

Fig. S1 Phylogenetic tree of 25 inbred rat strains for each of the 20 rat autosomes. Each tree was constructed by the neighbor-joining algorithm based on SNV data of all the coding regions of the designated chromosome. The scale bar at the bottom right indicates 1 nucleotide substitutions per 10,000 bases

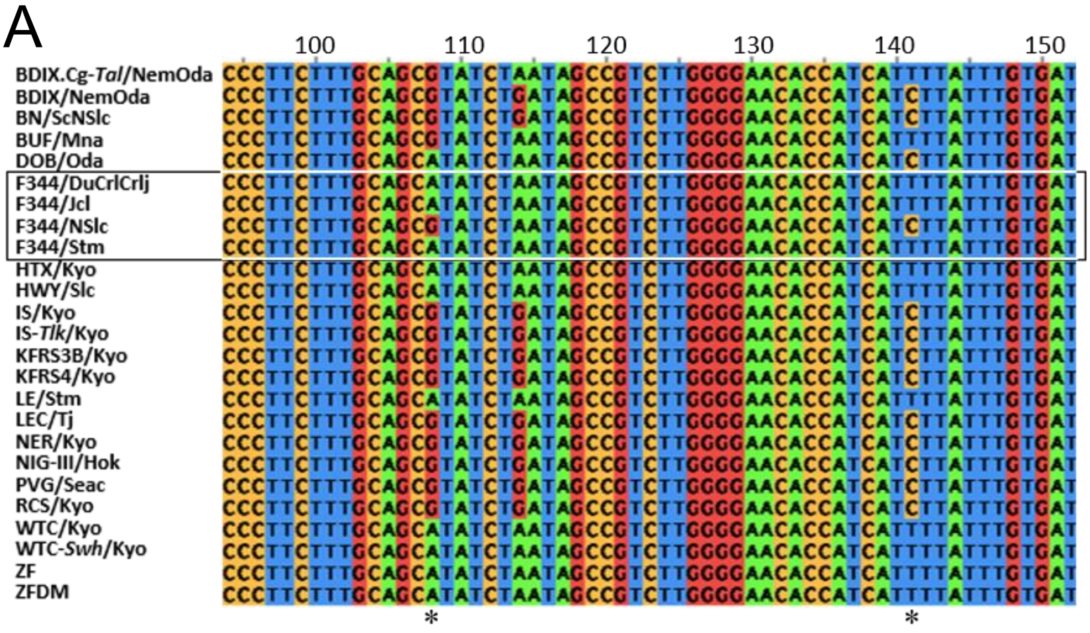

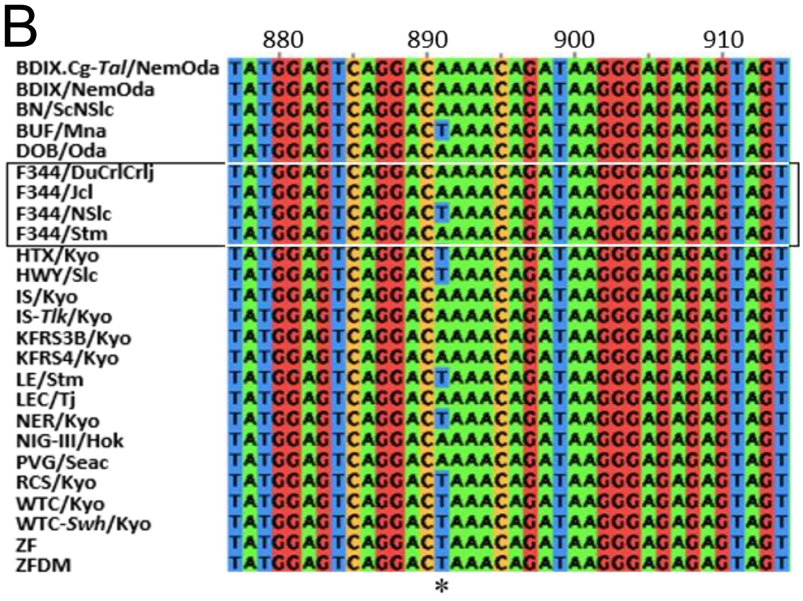

Fig. S2 Example of discordant sites in multiple sequence alignments of *Olr186* gene on 1q33. The box indicates the cluster of F344 substrains. The numbers above the alignment indicate the positions in mRNA. The asterisks indicate the discordant sites in the alignments. **a** Discordant sites observed in the position from 94 to 152 in the cluster of F344 substrains. **b** Discordant sites observed in the position from 877 to 914 in the cluster of F344 substrains

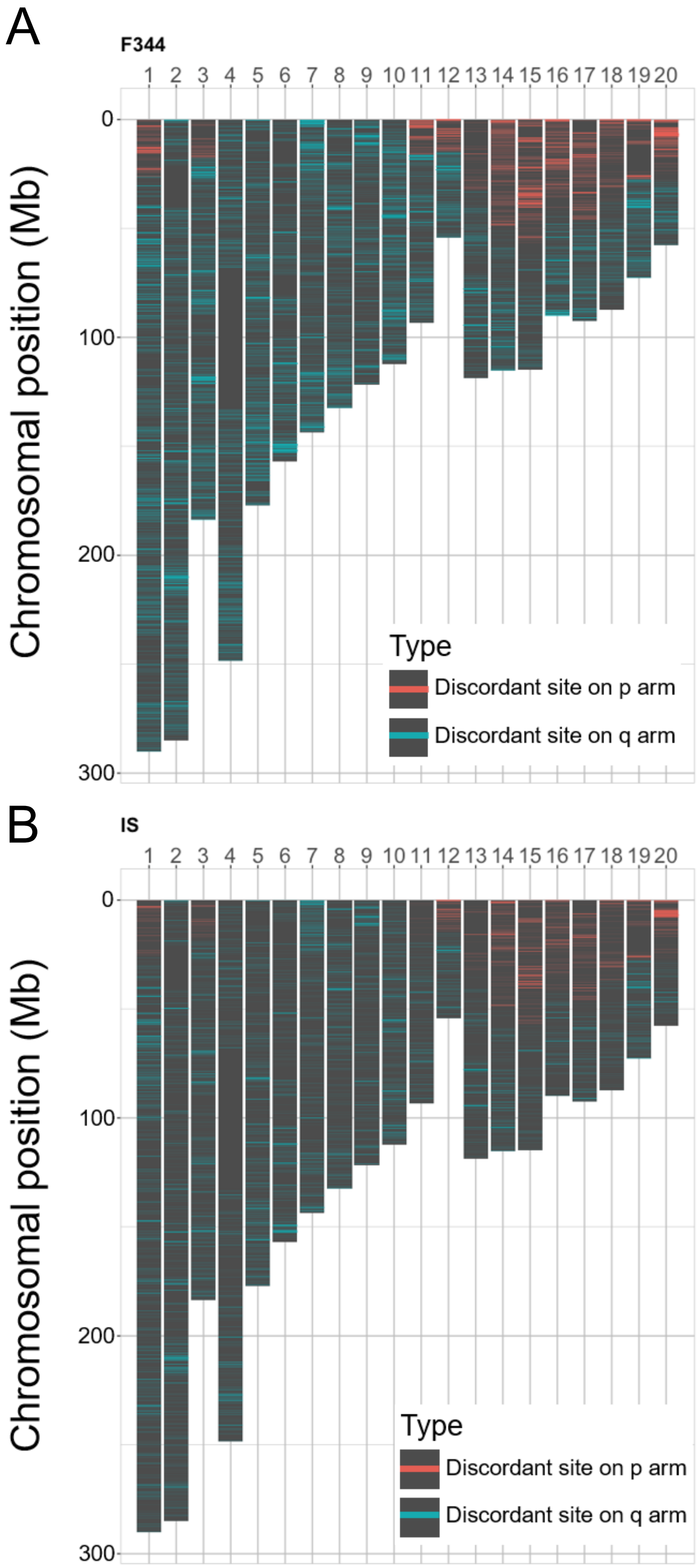

A
F344
1 2 3 4 5 6 7 8 9 10 11 12 13 14 15 16 17 18 19 20
Chromosomal position (Mb)
0
100
200
300
Type
Discordant site on p arm
Discordant site on q arm
B
IS
1 2 3 4 5 6 7 8 9 10 11 12 13 14 15 16 17 18 19 20
Chromosomal position (Mb)
0
100
200
300
Type
Discordant site on p arm
Discordant site on q arm

C

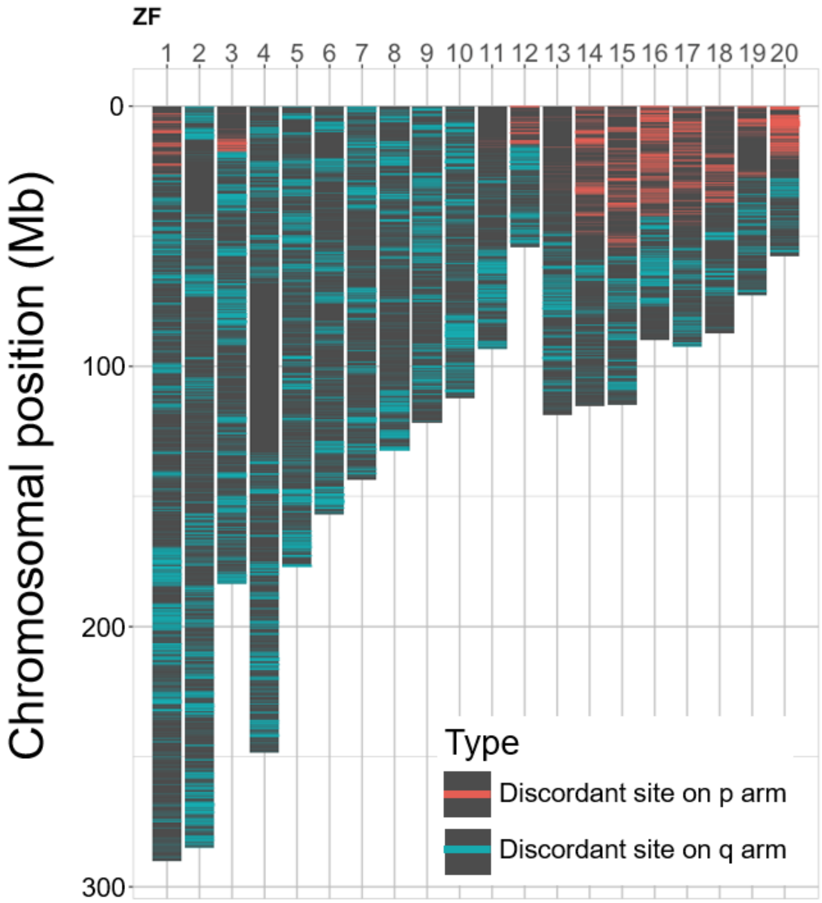

Fig. S3 Chromosomal distribution of discordant sites. Chromosomal distribution of discordant sites in autosomes of the clusters of (**a**) F344, (**b**) IS and (**c**) ZF substrains. The red and blue horizontal lines on the chromosomes represent discordant sites in the short (p) arm and long (q) arm, respectively

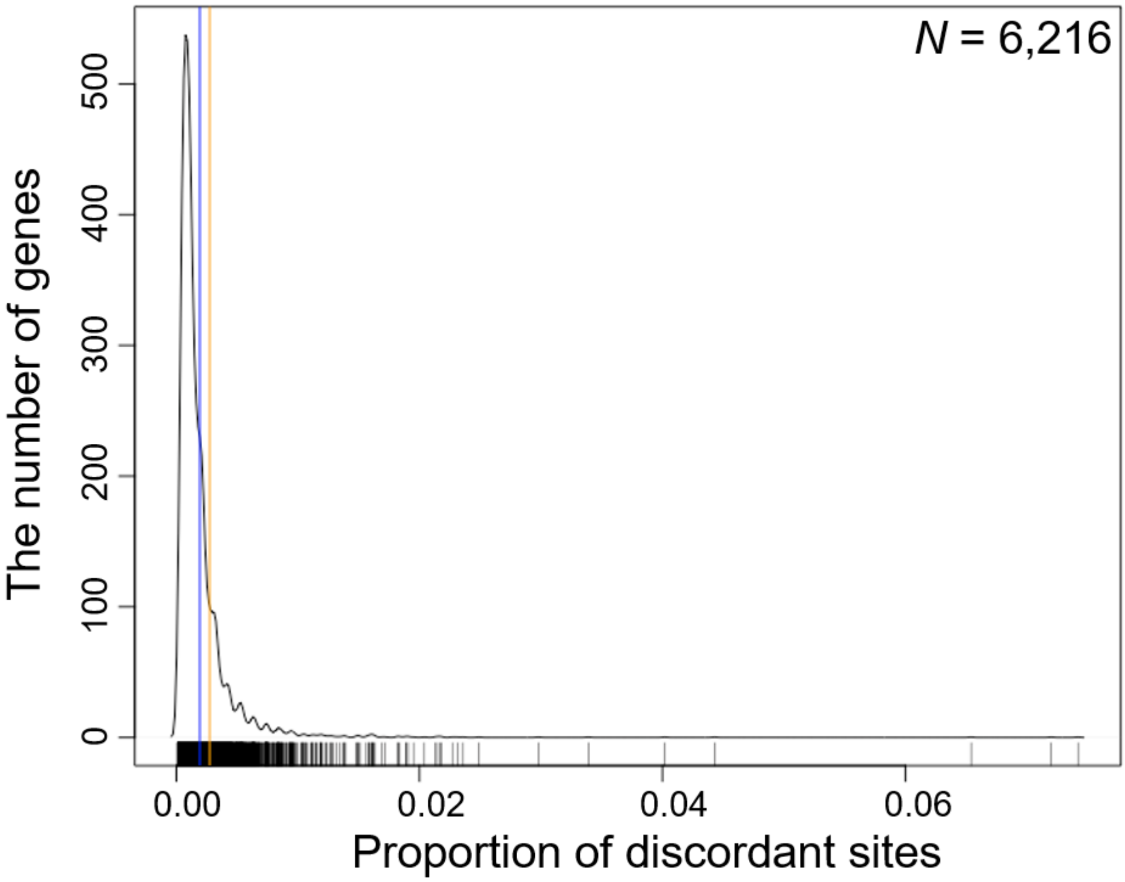

Fig. S4 Density distribution of proportion of discordant sites in each gene. The total number of the genes was 6,216. The orange and blue vertical lines denote, respectively, mean value and standard deviation value of the proportion of discordant sites. Proportion of discordant sites for a gene was calculated as the counts of discordant sites divided by the gene length

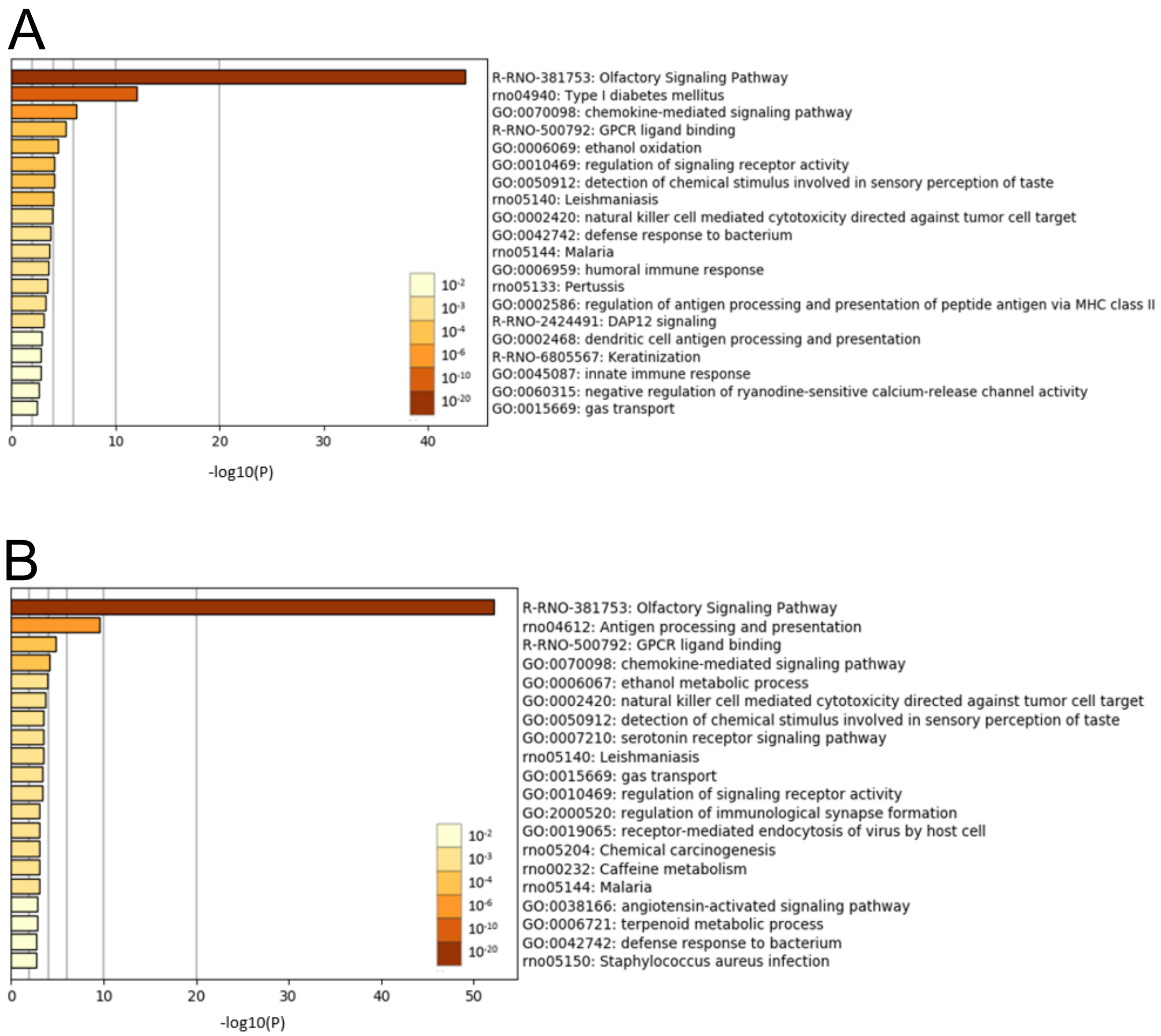

Fig. S5 Bar graphs of functional enrichment analysis by Metascape. The enrichment analysis was performed using the top (**a**) 1,176 genes and (**b**) 2,035 genes from the list of genes with discordant sites. The bars are colored by *P*-values. GO, Gene ontology; rno, KEGG Pathway for rats; R-RNO, Reactome gene sets for rats

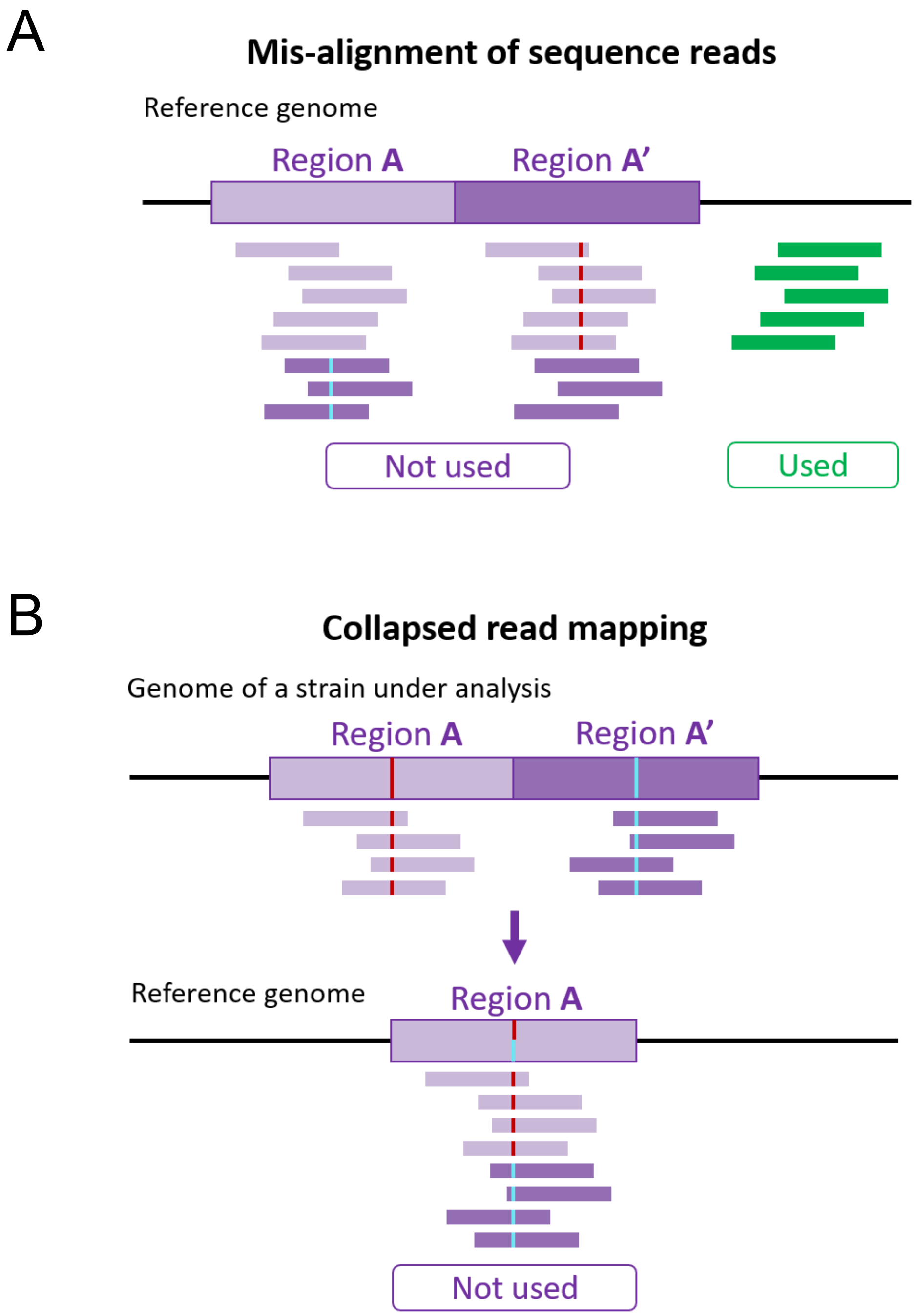

Fig. S6 Schematic view of possible alignment errors and their solutions. Black line indicates unique genomic region. A lavender box (region A) has duplicated to a purple box (region A'). They are highly similar genomic regions with each other. The bars below the boxes and the line are sequencing reads for those genomic regions. (a) Mis-alignment of sequencing reads in duplicated regions. To prevent false variant calling in such regions, we only used uniquely mapped reads for variant calling. (b) Collapsed read mapping. This can happen if the genome of a strain under analysis has a duplication, while the reference genome has no such duplication. In such a region, any differences between the duplicated regions result in heterozygous variants. To prevent such false variants, we only used homozygous variants for discordant site detection
